# Obese patients exhibit a greater enhancement in mental health-related quality of life compared to non-obese patients following thoracoscopic ablation of atrial fibrillation

**DOI:** 10.3389/fcvm.2025.1433790

**Published:** 2025-03-04

**Authors:** Eva R. Meulendijks, Manouck J. M. Roelofs, Tim A. C. de Vries, Robin Wesselink, Rushd F. M. Al-Shama, Wim-Jan P. van Boven, Antoine H. G. Driessen, Wouter R. Berger, Jonas S. S. G. de Jong, Joris R. de Groot

**Affiliations:** ^1^ Department of Clinical and Experimental Cardiology, and Department of Cardiothoracic Surgery, Amsterdam UMC, University of Amsterdam, Heart Center, Amsterdam, Netherlands; ^2^ Amsterdam Cardiovascular Sciences, Amsterdam, Netherlands; ^3^ Department of Cardiology, Rijnstate Ziekenhuis, Arnhem, Netherlands; ^4^ Department of Cardiology, OLVG, Amsterdam, Netherlands

**Keywords:** obesity, atrial fibillation, quality of life, ablation, thoracoscopic, SF36 health survey

## Abstract

**Background:**

Obesity is an important risk factor for atrial fibrillation (AF) development and progression. Furthermore, obesity reduces health-related quality of life (HRQoL), an essential indicator for treatment efficacy of AF ablation. Nevertheless, the extent to which obesity influences changes in HRQoL and the recurrence of AF following ablation, especially thoracoscopic AF ablation, remains largely unexplored.

**Aims:**

We assessed in obese vs. non-obese patients undergoing thoracoscopic AF ablation: (1) HRQoL upon ablation, (2) AF recurrence incidence, (3) the association between recurrence incidence and HRQoL.

**Methods & results:**

408 prospectively enrolled patients were included for analysis. Heart rhythm was systematically monitored during follow-up. AF recurrence was defined as any atrial tachyarrhythmia episode > 30 s. HRQoL and recurrence incidence were assessed for normal weight (BMI ≤ 24.9 kg/m^2^), overweight (25.0–29.9 kg/m^2^) and obese (≥30.0 kg/m^2^) patients. HRQoL was assessed at baseline and 1-year follow-up. Obese patients scored lower in pre-operative HRQoL across 6/8 subscales vs. non-obese patients (*p* < 0.01–0.05). While HRQoL increased in all patients, obese patients showed a trend towards an even greater improvement of mental HRQoL (*p* = 0.07) vs. non- obese patients. In obesity, mental HRQoL increased similarly for those with and without AF recurrence (*p* = 0.78), whereas in non-obese patients, AF recurrence was associated with less improved mental HRQoL (*p* = 0.03). AF recurrence at 1-year was similar between all weight groups (72.4%, 68.0%, 70.4%, *p* = 0.69).

**Conclusions:**

After thoracoscopic ablation, obese patients experience a comparable incidence of AF recurrence as non-obese patients. Interestingly, obese patients also exhibit a more significant enhancement in mental quality of life, regardless of whether AF recurs or not.

## Introduction

Atrial fibrillation (AF) is the most prevalent cardiac arrhythmia and is associated with a significant rise in morbidity and mortality (1). It is projected that the prevalence of AF will at least double by 2050 (2). Obesity accounts for nearly 60% of the increase in AF incidence (3). Every unit increase in body mass index (BMI) leads to an additional risk of AF between 3.5% and 5.3% (4). Not only a weight within the obesity range (3–8), but also weight fluctuation increases the risk of AF (9).

Furthermore, obesity may contribute to progression from paroxysmal to persistent AF (10). Also, obesity has been associated with higher AF recurrence in patients undergoing catheter ablation (CA) (11, 12). However, several studies showed no effect of obesity on efficacy, particularly after adjusting for confounders (13). Therefore impact of obesity of AF ablation remains uncertain (14, 15). Factors such as left atrial enlargement can contribute to more AF recurrences in obese patients (12).

Both AF and obesity are associated with a reduced health-related quality of life (HRQoL): AF due to severity of symptoms related to AF (16), while obesity is associated with lower HRQoL due to its impact on mental health, which in turn affects all facets of a patient's HRQoL (17). HRQoL is a subjective evaluation of symptoms, functional status and patient's health perceptions, measured by using standardized questionnaires (for example Short Form 36 questionnaires) (18).

For patients with symptomatic AF who do not respond to antiarrhythmic drugs, minimally invasive thoracoscopic ablation is a viable alternative to CA (19–21). The primary goal of thoracoscopic ablation is to improve AF-related symptoms; therefore, the improvement of HRQoL serves as an essential indicator of treatment efficacy. However, the impact of obesity on HRQoL change following thoracoscopic AF ablation is not fully understood, neither is its influence on the outcome of thoracoscopic AF ablation (1, 7). Gaining a better understanding of these associations may benefit personalized AF treatment and aid patient selection for invasive AF treatment in the future.

We assessed in obese vs. non-obese patients undergoing thoracoscopic AF ablation: (1) HRQoL change upon ablation, (2) AF recurrence incidence, (3) the association between recurrence and HRQoL.

## Methods

### Study design and setting

Patients from this study are enrolled in the Markers for Determining Atrial Remodeling in Patients with Atrial Fibrillation (MARK-AF) registry [CCMO Registry, NL50069.018.14, Amsterdam: Academic Medical Center (The Netherlands)]. MARK-AF is a single-center, prospective cohort study of patients undergoing thoracoscopic ablation for AF at a tertiary academic hospital (the Amsterdam UMC, Academic Medical Centre). Paroxysmal AF was defined as AF lasting less than 7 days, while persistent AF lasted more than 7 days, according to the HRS/EHRA guidelines (22). Patients enrolled in this study had symptomatic, advanced AF, defined as usually persistent, with enlarged left atria with or without a history of failed CA (20), and were refractory to at least one trial of class I or III antiarrhythmic drugs (19). The study conformed to the Declaration of Helsinki and all participants provided written informed consent.

### Patient selection and data collection

**Figure 1:**
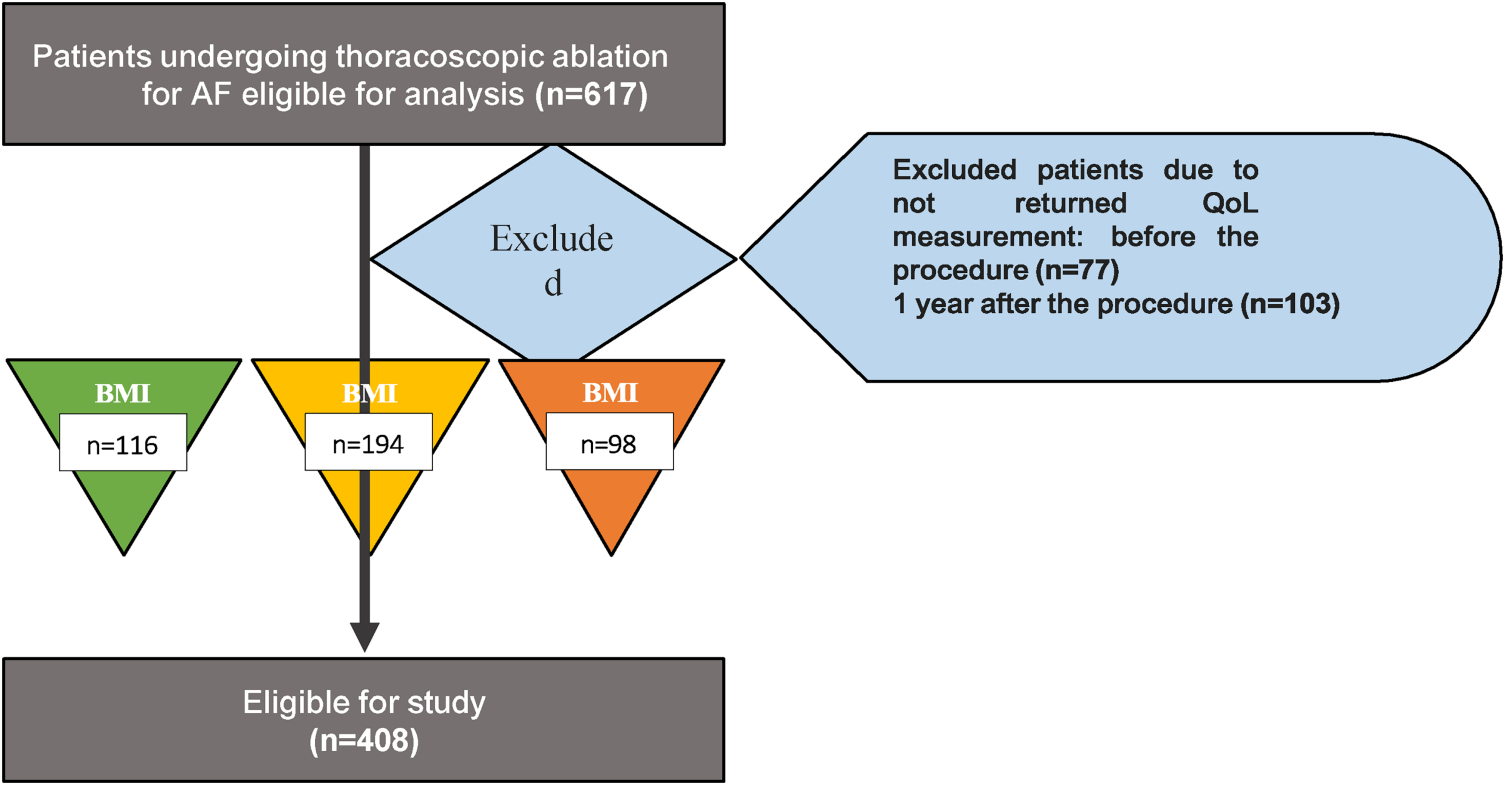
Flowchart study. Study flow chart. Eventually 408 patients were eligible for study. Patients were divided based on their BMI at baseline in three different groups: normal weight (BMI <25), overweight (BMI 25-30) and obese group (BMI ≥30). BMI, body mass index.

For the current analysis, patients were enrolled in MARK AF from September 2009 through March 2020. Patient characteristics and HRQoL-questionnaires were collected at baseline and at 12 months follow-up. Patients in whom BMI or HRQoL were not recorded at baseline or 12 months of follow-up were excluded from this analysis (*n* = 209) (33.9%) (Figure 1).

HRQoL was assessed using the Short Form 36 (SF-36) questionnaire, which was self-administered. The SF-36 is a widely used HRQoL survey instrument consisting of 36 questions. It includes eight health concepts using multi-item scales including physical function (PF), social functioning (SF), role physical (RP), role emotional (RE), mental health (MH), vitality (VT), bodily pain (BP) and general health (GH). In addition to these eight subscales, the SF-36 includes two summary scale scores – physical component summary (PCS) and mental component summary (MCS). Both summary scores are calculated by the sum of all eight SF-36 health concepts multiplied by their respective physical or mental scoring coefficient.

The score of each subscale of the SF-36 was calculated as the mean of items. All scores were recoded to scores between 0 and 100 (with 100 being the best possible health) (23). All eight subscales were computed into Z-scores using a dataset displaying the HRQoL in the general Dutch population (24). PCS scores was constructed by multiplying each SF-36 scale Z-score by its respective physical factor scoring coefficient and summing the eight products. Similarly, MCS scores were created by multiplying each SF-36 scale Z-score by its respective mental factor score coefficient and summing the products. We used radar charts to visualize all subscales in different BMI groups (18).

### Definition of key variables

Patients were categorized in three groups at baseline according to the WHO's BMI definitions (25): normal weight (BMI 18.5–24.9 kg/m^2^); overweight (BMI 25.0–29.9 kg/m^2^); and obese (BMI ≥ 30.0 kg/m^2^). HRQoL and AF recurrence incidence were assessed for each group.

AF recurrence was defined as any episode of AF, atrial flutter or tachycardia documented on ECG or Holter registration after a blanking period of three months, but within one year after the ablation procedure, lasting longer than 30 s consecutively (22).

### Statistical analysis

Continuous values are described as mean ± standard error (SE) or standard deviation (SD), or median and interquartile range (IQR) for normal and non-normally distributed variables respectively. Ordinal variables were reported as either median and IQR or as number and percentage of patients. Nominal variables were expressed as the number and percentage of subjects. Normality of continuous variables was visually assessed using histograms and boxplots and tested using the Kolmogorov-Smirnov test. When visual estimation was discrepant with the Kolmogorov-Smirnov test, a concluding assessment was performed by another co-author. Differences in ratio or interval variables between the BMI groups at baseline were assessed using the one-way ANOVA test for normally distributed data, the Kruskal- Wallis test for non-normally distributed ratio or interval data or ordinal variables, and with the Chi- square tests for nominal data. Change in HRQoL in each group was compared using the Wilcoxon Rank Test.

To assess the difference in recurrence incidences for patients in the different BMI groups and obese vs. non-obese groups, we divided the patients in those who did not experience an AF recurrence and those who did. Change in HRQoL was compared between the two groups by using the unpaired t- test in normal distributed data and the Mann-Whitney test in non-normally distributed data.

We used linear mixed models to estimate the effect of different determinants on the change in PCS or MCS between the baseline and follow-up visit, as further explained in Supplementary Table 4.

A two-tailed *p*-value of <0.05 was considered statistically significant for all analyses. All statistical analyses were performed using “IBM SPSS Statistics for Windows, version 26 (IBM Corp., Armonk, N.Y., USA)” and ’Studio R version 4.3.0. (R Foundation for Statistical Computing, Vienna, Austria)'.

## Results

### Patient characteristics

We included four hundred eight patients who underwent thoracoscopic ablation for AF and completed baseline and 1-year follow-up SF-36 data (Figure 1). Of these patients, 116 (28.4%) had a normal weight (BMI 21.3 ± 1.4 kg/m^2^), 194 (47.5%) were overweight (BMI 27.2 ± 1.4 kg/m^2^) and 98 (24.0%) were obese (BMI 32.8 ± 2.2 kg/m^2^). Obese patients showed a trend toward a younger age compared to patients with overweight and normal weight (58.9 ± 9.5 vs. 60.5 ± 8.3 and 61.6 ± 9.1 years, respectively, *p* = 0.07). Obese patients had a significantly higher prevalence of hypertension and diabetes compared to overweight and normal patients (hypertension: 55.1%, 30.2%, and 47.9%, respectively, *p* < 0.01, and diabetes mellitus: 12.2% vs. 4.6% or 1.6%, *p* < 0.01) (Table 1).

**Table 1:** Baseline characteristics for different BMI groups.

Baseline patient characteristics		Total group	BMI groups			*P*-value
			BMI <25	BMI 25–30	BMI ≥30	
Patients (*n*, %)		408	116 (28.4%)	194 (47.5%)	98 (24.0%)	
Age (y) (±SD)		61.6 ± 1.1	61.6 ± 9.14	60.5 ± 8.3	58.9 ± 8.5	0.07
Female (*n*, %)		107 (26.2%)	41 (35.3%)	40 (20.6%)	26 (26.5%)	**0** **.** **02**
BMI (kg/m^2^) (±SD)		27.4 ± 0.4	23.1 ± 1,4 (18.0–24.9)	27.2 ± 1.4 (25.0–29.9)	32.8 ± 2.2 (30.0–39.3)	**<0** **.** **01**
Type AF (*n*, %)	Paroxysmal	160 (39.2%)	55 (47.4%)	73 (37.6%)	32 (32.7%)	0.17
	Persistent	248 (60.8%)	61 (52.6%)	121 (62.4%)	66 (67.3%)	
AF duration (y) [IQR]		4 (6)	4 (4)	4 (6)	5 (7)	0.95
Myocardial infarction (*n*, %)		23 (5.6%)	5 (4.3%)	13 (6.7%)	5 (5.1%)	0.65
Risk factors (*n*, %)	CHD	23 (5.6%)	5 (4.3%)	10 (5.2%)	8 (8.2%)	0.44
	Hypertension	182 (44.6%)	35 (30.2%)	93 (47.9%)	54 (55.1%)	**<0**.**01**
	>65 years	145 (35.5%)	47 (40.5%)	74 (38.1%)	24 (24.5%)	**0**.**03**
	>75 years	9 (2.2%)	4 (3.5%)	4 (2.1%)	1 (1.0%)	0.48
	DM	24 (5.9%)	3 (2.6%)	9 (4.6%)	12 (12.2%)	**<0**.**01**
	Stroke	33 (8.1%)	8 (6.9%)	16 (8.2%)	9 (9.2%)	0.82
	Vascular disease	57 (14.0%)	14 (12.1%)	29 (14.9%)	14 (14.3%)	0.77
CHA₂DS₂-VASc- score (*n*, %)	0 (*n*, %)	102 (25.0%)	32 (27.6%)	49 (25.3%)	21 (21.4%)	0.58
	1 (*n*, %)	134 (32.8%)	40 (34.5%)	60 (30.9%)	34 (34.7%)	0.74
	≥2 (*n*, %)	172 (42.2%)	44 (37.9%)	85 (43.8%)	43 (43.9%)	0.55
CHA₂DS₂-VASc-score [IQR]		2 (2)	2 (4)	2 (2)	1 (1)	0.18
Anti-arrhythmic medication use	(*n*, %)	394 (96.6%)	111 (95.7%)	190 (97.9%)	93 (94.9%)	0.30
Heart rate (bpm) (±SD)		72.2 ± 19.3	71.6 ± 21.3	72.9 ± 21.0	77.9 ± 21.0	0.08
Echocardiographic parameters (mean ± SD)	LAVI (ml/m^2^)	42.3 ± 1.41	41.7 ± 12.5	42.3 ± 13.5	40.8 ± 9.8	0.67
	LVEF (%)	53.0 ± 1.1	56.2 ± 6.8	52.1 ± 9.8	50.2 ± 10.0	0.06
NT-pro-BNP (pg/ml) [IQR]		456 (638.3)	431 (599.0)	456 (787.8)	515 (390.0)	0.36

Baseline characteristics of total group and three BMI groups; normal weight (BMI <25), overweight (BMI 25–30), obese (BMI ≥30).

AF, atrial fibrillation; BMI, body mass index; CHD, congestive heart disease; DM, diabetes mellitus; LAVI, left atrial volume index; LVEF, left ventricle ejection fraction.

Bold values denote the significant *P*-values.

### Obesity is associated with lower baseline quality of life

**Figure 2:**
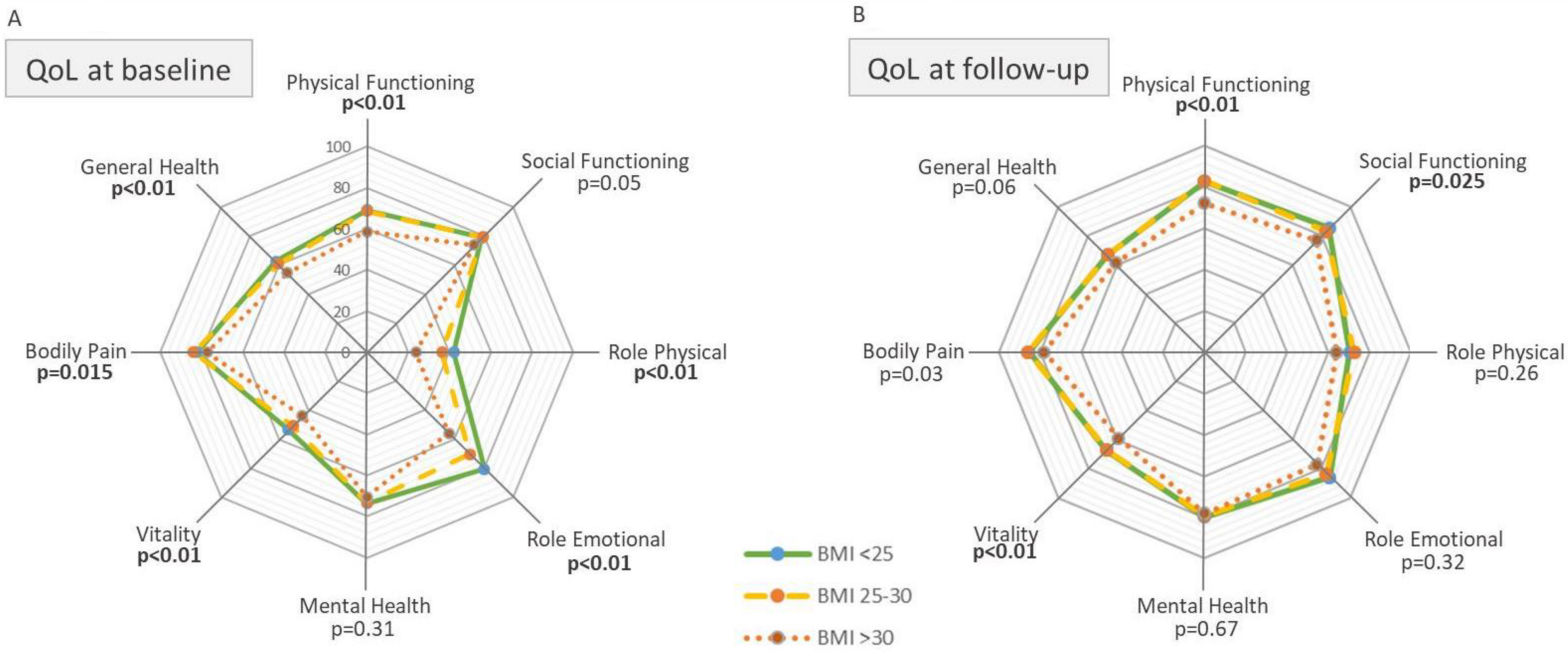
Quality of life before and after thoracoscopic AF ablation in normal, overweight and obese patients. The radar charts represent the eight quality of life (QoL) domains at baseline **(A)** and follow-up **(B)**, for each BMI group. From inside out colored lines represent the obese, overweight and normal weight groups, respectively. The center of the Radar chart displays a QoL-score of 0 (=low) and the outer edge a score of 100 (=high). *P*-values represent significant difference in QoL-score between BMI groups. All *p*-values in bold are significant (*p* < 0.05). For definitions of each individual domain please refer to “definitions” in the “Methods” section.

At baseline, obese AF patients had lower HRQoL in six of eight SF-36 subscales (PF, RP, RE, VT, BP, GH) compared to non-obese patients (Figure 2A). This was apparent in the comparison between obese AF patients vs. normal weight AF patients, as well as obese AF patients vs. overweight AF patients (Supplementary Table 1)*.* Before thoracoscopic ablation, PCS and MCS were significantly lower in obese compared to normal weight patients (PCS: 41.7 ± 1.0 vs. 45.3 ± 0.6 (*p* = 0.02), and 44.8 ± 0.8 (*p* < 0.01); MCS: 43.1 ± 1.2 vs. 45.9 ± 0.8 (*p* < 0.01), and 47.7 ± 1.0 (*p* < 0.05), for obese, overweight and normal weight patients, respectively) (Supplementary Table 1).

### Change in quality of life after thoracoscopic Af ablation is independent of obesity status

Scores of six out of eight SF-subscales were significantly higher after the procedure [PF: 13.9 ± 1.1, SF: 4.7 ± 1.8, RP: 34.8 ± 2.5, RE: 12.6 ± 2.2, MH: 6.6 ± 0.7, VT: 15.5 ± 1.0, and GH: 5.7 ± 0.9, (*p* < 0.01)], and were equal for all BMI groups. Bodily pain did not improve (*p* = 0.10) (Supplementary Tables 1, 2; Figure 2B). “Health change” scores were similar: 81.0 ± 2.3, 80.0 ± 1.8 and 81.0 ± 2.7; for normal weight, overweight and obese patients, respectively, (*p* = 0.72) (Supplementary Table 1).

**Figure 3:**
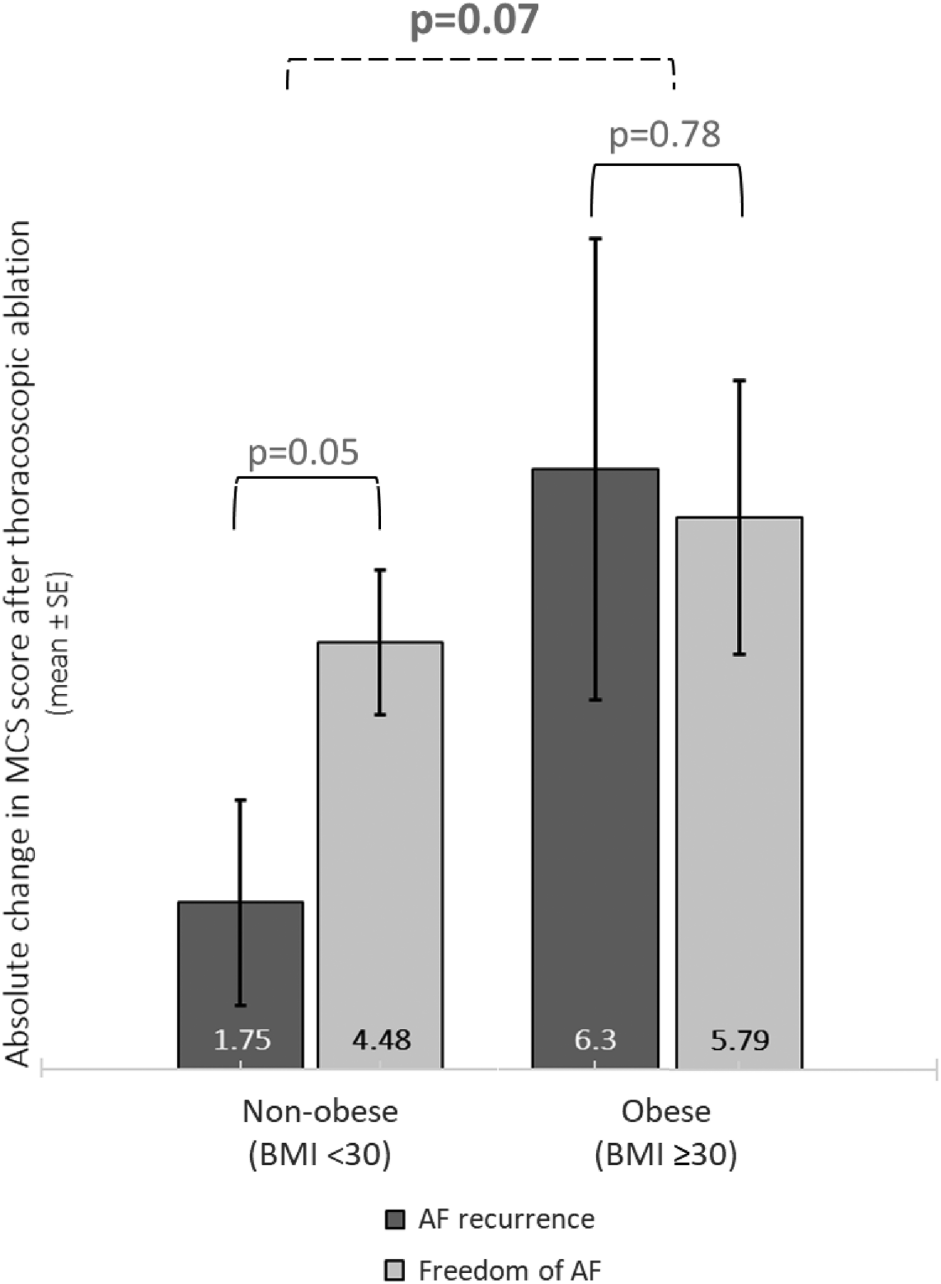
Improvement of mental quality of life in obese and non-obese patients according to the state of AF recurrence. Bar charts represent the change of MCS score in non-obese vs. obese patients, according to AF recurrence status. Mean ± SE. SE = standard error.

The SF-subscale Role Emotional (RE) improved more in obese patients compared to non-obese patients (obesity: 22.0 ± 5.0, overweight: 12.6 ± 3.1, normal: 4.9 ± 3.8, *p* < 0.05). Improvement of the other SF-subscales did not significantly differ between BMI groups (Supplementary Figures 1A–C; Supplementary Table 2). Both PCS and MCS improved in all patients following thoracoscopic ablation (PCS: 4.7 ± 0.5, MCS: 4.2 ± 0.6, *p* < 0.01). Subgroup analysis showed a trend for a stronger increase in MCS score in obese compared to overweight and normal weight patients (6.1 ± 1.2, 3.9 ± 0.8, 3.2 ± 1.0, *p* = 0.1, and *p* = 0.09) (Supplementary Table 2). This trend remained when comparing the 98 obese (6.1 ± 1.2) with the 310 non-obese patients (3.7 ± 0.6) (*p* = 0.07) (Figure 3).

### Atrial fibrillation recurrence affects HRQoL differently in obese compared to non-obese patients

Two hundred eighty-five (69.9%) patients were free from AF recurrence after one year of follow-up. Recurrence incidences were similar between obese and non-obese patients (*p* = 0.69) (Table 2).

**Table 2:** AF recurrence status for obese and non-obese patients.

Outcome after ablation	Total group	BMI <25	BMI 25–30	BMI ≥30	*p*-value
Presence of AF recurrence at 1 year follow-up					
Freedom of AF	285 (69.9%)	84 (72.4%)	132 (68.0%)	69 (70.4%)	0.69
AF recurrences	123 (30.1%)	32 (27.6%)	62 (32.0%)	29 (29.6%)	

Table shows AF recurrence status of all patients 1 year after thoracoscopic ablation (*n*, %).

Patients without vs. those with recurrence at 1 year follow-up had a greater improvement in HRQoL [PF: 16.8 ± 1.3 vs. 7.8 ± 2.1, RP: 40.5 ± 3.0 vs. 20.7 ± 4.6, MH: 7.8 ± 0.9 vs. 3.2 ± 1.4, VT: 17.2 ± 1.2 vs. 10.6 ± 1.7, GH: 9.5 ± 1.1 vs. −2.9 ± 1.6, PCS: 6.0 ± 0.5 vs. 1.6 ± 0.8, and health change 85.9 ± 1.8 vs. 67.6 ± 3.1; (all *p* < 0.01)]. The QoL differences between patients without vs. those with recurrence were more pronounced in SF-subscales of overweight than obese patients (overweight: PF: 17.0 ± 1.7 vs. 9.4 ± 2.5, RP: 42.9 ± 4.3 vs. 21.3 ± 6.2, VT: 19.2 ± 1.7 vs. 10.9 ± 2.2, GH: 9.4 ± 1.5 vs. −1.2 ± 2.2, PCS: 5.9 ± 0.8 vs. 2.0 ± 1.0, and health change: 85.2 ± 2.6 vs. 69.0 ± 3.7, (all *p* < 0.01); SF: 5.8 ± 3.4 vs. −1.2 ± 4.1, RE: 16.5 ± 3.9 vs. 4.4 ± 4.8, MH: 7.2 ± 1.3 vs. 3.8 ± 1.6, BP: 2.8 ± 1.8 vs. −2.9 ± 2.5 (all *p* < 0.05), obese: GH: 10.7 ± 2.5 vs. −2.6 ± 3.9, (*p* < 0.01), PCS: 5.8 ± 1.2 vs. 1.2 ± 1.9, and health change: 88.0 ± 3.8 vs. 63.0 ± 7.4, (*p* < 0.05)) (Supplementary Table 3).

Obese AF patients had the greatest absolute improvement of MCS score, irrespective of AF recurrence (5.8 ± 1.4 vs. 6.3 ± 2.4 in AF freedom and AF recurrence, respectively; *p* = 0.78). Normal weight patients with freedom of AF had a numerically larger absolute improvement of MCS score compared to patients with AF recurrence (4.0 ± 1.1 vs. 1.0 ± 2.0, respectively; *p* = 0.21), while there was a trend in the overweight group (4.8 ± 1.0 vs. 2.1 ± 1.3, in patients with AF freedom and AF recurrence respectively; *p* = 0.06) (Supplementary Table 3). Non-obese patients with freedom of AF had a significantly larger improvement of MCS scores compared to non-obese patients with AF recurrence (4.48 ± 0.78 vs. 1.08 ± 1.75, *p* = 0.05) (Table 3; Figure 3).

**Table 3:** Physical and mental component summary scores in obese and non-obese patients according to recurrence state.

PCS and MCS scores per BMI group		Change in quality of life						
		AF Recurrence			Freedom of AF			Score change in patients with vs. without AF recurrence *p*-value
		Mean change	SE	Within group *p*-value	Mean change	SE	Within group *p*-value	
Total group	PCS	1.56	0.77	**0**.**05**	6.00	0.54	**<0**.**01**	**<0**.**01**
	MCS	2.80	1.01	**<0**.**01**	4.80	0.67	**<0**.**01**	0.07
BMI <30	PCS	1.66	0.83	0.05	6.13	0.60	**<0**.**01**	**<0**.**01**
	MCS	1.75	1.08	0.11	4.48	0.76	**<0**.**01**	**0**.**03**
BMI ≥30	PCS	1.20	1.90	0.53	5.75	1.21	**<0**.**01**	**0**.**05**
	MCS	6.30	2.42	**0**.**02**	5.82	1.43	**<0**.**01**	0.78

Table shows the difference in change in physical component summary (PCS) and mental component summary (MCS) scores in patients with AF recurrence vs. freedom of AF for the total group and for obese/non-obese patients. All *p*-values in bold are significant (*p* < 0.05).

In our linear mixed models, significant determinants for change in PCS were: AF recurrence at 1 year of follow-up, CHADsVASc score of 0 (vs. 1 or more) and pro-BNP (Supplementary Table 4).

Interaction terms were not interpretable individually for BMI, however Supplementary Figure 2 shows that the higher the BMI score is, the lower the change in PCS is. Significant determinants for change in MCS were CHADsVASc score of ≥2 (vs. < 2), hypertension, diabetes and total number of anti- arrhythmic medication at baseline (Supplementary Table 4).

In the sensitivity analysis, excluding patients without HRQoL forms at one timepoint did not alter PCS and MCS scores (Supplementary Table 5). Other potential confounders (change in weight or BMI class during follow-up, or separating the population based on quartiles and WHO classes) were assessed and did not change the results above.

## Discussion

Primarily, we assessed the impact of obesity on the improvement of HRQoL after thoracoscopic AF ablation. Secondly we assessed the relation between obesity and AF recurrence. At baseline, AF patients who were obese exhibited a notably lower HRQoL compared to AF patients with normal weight. Obese patients showed a greater improvement of mental HRQoL score after thoracoscopic ablation, whereas other HRQoL-subscales increased similarly, compared to normal weight patients. Furthermore, among AF patients of normal weight, there was less improvement in mental HRQoL in those with, as opposed to those without AF recurrence. For obese patients, the improvement in mental HRQoL was similar for those with and without AF recurrence.

Pre-procedural HRQoL was lower in obese patients compared to normal weight patients, which is consistent with earlier work of Zhuang et al. (26). This may be due to reduced physical HRQoL caused by pain and decreased mobility (27, 28). Moreover, social discrimination and poorer body image may have reduced mental HRQoL (17, 19). Furthermore, both obesity and psychological stress are associated with reduced bioavailability of BDNF, brain-derived neurotrophic factor (BDNF) (29). BDNF reflects brain health as it support neuronal cells in managing stress (30, 31). At baseline it may be that obese subjects experience a reduced local pool of BDNF, both at the brain and heart levels, as shown experimentally in mice (32). Furthermore, it may be possible that thoracoscopic AF ablation reduces psychological stress and subsequently compensates for the loss of BDNF bioavailability in obese patients, contributing to a higher increase of mental QOL compared to non-obese. Generally, aside from mental health, HRQoL score increased equally in obese and non-obese patients. This suggests that thoracoscopic AF ablation had a consistent positive impact on HRQoL, irrespective of the patient's obesity status. Similarly, Cha et al. (33). Showed an equal HRQoL improvement in normal weight, overweight and obese patients after CA of AF. However, Mohanty et al. (34). Generally showed a greater improvement of HRQoL (but not MH, SF and BP) after CA in patients with a high-BMI (≥25 kg/m^2^) versus normal-BMI (<25 kg/m^2^). They also showed that HRQoL improved in patients with high but not low-BMI. This could be due to the relatively high baseline physical HRQoL scores of non-obese AF patients in the study of Mohanty (34); The high baseline HRQoL establishes a narrower margin for potential improvement in HRQoL.

In our study, obese patients showed a greater improvement of mental summary score (MCS) upon thoracoscopic ablation than normal-BMI patients. We hypothesize that this may be attributable to a relative greater reduction in duration of AF episodes (AF burden) in obese vs. non-obese patients. Indeed, there was a trend towards a higher ratio of patients with persistent to paroxysmal AF in patients with obesity vs. a normal BMI. Irrespective of the cause, the lower pre-operative mental

HRQoL among obese vs. non-obese patients may have provided a larger margin for improvement after treatment. Differences in patient characteristics may explain that obese (and overweight) patients experience a more pronounced improvement in HRQoL vs. normal weight patients. In our cohort, obese vs. non-obese patients are younger and more often men. A younger age has been associated with poorer mental health before ablation (35–37). Aging of relatively young patients during follow-up may have a greater impact on improvement of HRQoL than in older patients. Female AF patients have been reported to suffer from a significantly lower HRQoL and a higher symptom burden than male AF patients (36–39). However, the linear mixed model analyses (Supplementary Table 4) showed that age and sex were not associated with changes in HRQoL.

We found that the proportion of patients with AF recurrence was similar between obese and non-obese patients, oppose to several CA studies showing that obesity increased the risk of AF recurrence (4). In those studies, a BMI exceeding 35.0 kg/m^2^ significantly influenced a 5-year AF recurrence (40). Yet, for those with long-standing persistent AF, obesity did not increase the risk on AF recurrence (40). Importantly, our cohort includes patients with advanced AF, i.e., patients with mostly persistent AF, an enlarged left atrium, and/or a prior failed CA (41). The substrate complexity in our cohort may be comparable to that in patients with long-standing persistent AF. One might speculate that the advanced remodeling stage could overshadow any remodeling effects caused by obesity, potentially through comorbidities in these patients that are associated with remodeling, such as hypertension or diabetes. On the other hand, hypertension and diabetes could be aggravated by presence or progression of obesity.

MCS scores increased in all patients after thoracoscopic ablation. However, in non-obese patients, those with recurrence of AF showed less improvement in MCS scores than those without. Conversely, in obese patients, MCS scores improved equally in patients with and without AF recurrence. We speculate that different factors could underlie this finding. First, obese compared to non-obese patients may experience fewer symptoms after ablation compared to before procedure. In general, obese patients have more co-morbidities (6, 42), more persistent AF (7, 42), and a lower HRQoL (17, 26–28, 33, 34, 43), than non-obese patients. Secondly, it is plausible that obese patients with AF possess more effective coping mechanisms compared to non-obese patients due to the presence of comorbidities. Therefore, if normal and overweight patients have a higher HRQoL at baseline, AF recurrence could be experienced as a larger drawback and a lower post-procedural mental HRQoL. Specifically, a BMI exceeding 35.0 kg/m^2^ significantly influenced a 5-year AF recurrence (40). Yet, for those with long-standing persistent AF, obesity did not increase the risk on AF recurrence (40).

By institutional consensus, catheter or thoracoscopic AF ablation is discouraged in morbidly obese patients (BMI ≥35.0 kg/m^2^). In this study, only 20 patients had a BMI ≥ 35.0 kg/m^2^; therefore, we do not know whether our results apply to morbidly obese patients. Given the results reported here, a prospective study including morbidly obese patients would be of interest. Such a study could outline whether (thoracoscopic) ablation is feasible in morbidly obese advanced AF patients and whether the increased risk of (respiratory) complications is balanced by an increase in HRQoL.

### Strengths and limitations

Despites the single-center design, this is currently the largest study investigating the effect of obesity on HRQoL in AF patients, allowing for HRQoL comparisons across normal weight, overweight and obesity upon thoracoscopic ablation.

In our study, patients answered HRQoL questions in the privacy of their homes without interference from healthcare professionals. This facilitated us in attaining a relatively unbiased assessment of patients' HRQoL. The SF-36 HRQoL questionnaire is a generic tool. Inherent to it, questions are not specified towards individual conditions such as obesity or AF (17). Therefore, the SF-36 makes it challenging to differentiate the parts of HRQoL allocated to AF or to obesity. However, as all patients, both obese and non-obese patients, had AF as primary diagnosis, we could dissect the contribution of obesity on HRQoL.

Anthropometrics were systematically measured at the policlinics, limiting measurement errors. The use of BMI as an obesity metric has been criticized for its inadequacy in measuring adiposity (44). The waist-to-hip ratio (WHR), focusing on abdominal, rather than overall body fat, emerges as a viable alternative to measuring adiposity. Our prior research indicated that both WHR and BMI serve as indicators for an elevated risk of developing new-onset AF (45). Despite its criticisms, BMI remains the golden standard for body composition assessment.

As continuous rhythm monitoring was not performed, the reported number of AF recurrences may be an underestimation. However, the systematically, regularly performed follow-up visits with Holter- monitoring are more stringent than current recommendations (26). Additionally, we postulate that asymptomatic AF recurrences occur similarly in obese and non-obese patients and are less likely to substantially affect general HRQoL.

AF recurrence is a dichotomous outcome, ignoring the number of AF recurrence episodes. A lower number of AF episodes in obese compared to non-obese patients may explain the different effects of recurrence on HRQoL. However, the number of registered episodes was similar for the AF recurrence patients in the obese and non-obese groups.

## Conclusion

Obese patients with AF have a lower health-related quality of life compared to non-obese patients. Nevertheless, after thoracoscopic ablation, obese patients experience a greater enhancement in mental quality of life than non-obese patients, irrespective of AF recurrence status. Notably, AF recurrence was similar between obese and non-obese patients.

### Clinical implications

Our study demonstrates that thoracoscopic ablation, in contrast to previous findings on catheter ablation, carries a similarly low AF recurrence rate for both obese and non-obese patients. Furthermore, obese patients may experience a more substantial improvement in mental health-related quality of life following thoracoscopic AF ablation. These findings may prompt a discussion on the feasibility of (thoracoscopic) ablation in individuals with morbid obesity and advanced AF.

## Data Availability

The datasets presented in this study can be found in online repositories. The names of the repository/repositories and accession number(s) can be found in the article/Supplementary Material.
