# Supplementary material for: Obese patients exhibit a greater enhancement in mental health-related quality of life compared to non-obese patients following thoracoscopic ablation of atrial fibrillation
Source: Front Cardiovasc Med. 2025 Mar 4;12:1433790. doi: 10.3389/fcvm.2025.1433790 (PMC11914143; doi:10.3389/fcvm.2025.1433790)
Supplement: Supplementary file 1 [file Datasheet1.docx]

**Supplementary tables & figures of the manuscript:**

**Obese patients exhibit a greater enhancement in mental health-related quality of life compared to non-obese patients following thoracoscopic ablation of atrial fibrillation**

**Supplementary Figure 1: Quality of life before and after VATS-PVI in normal, overweight and obese patients**


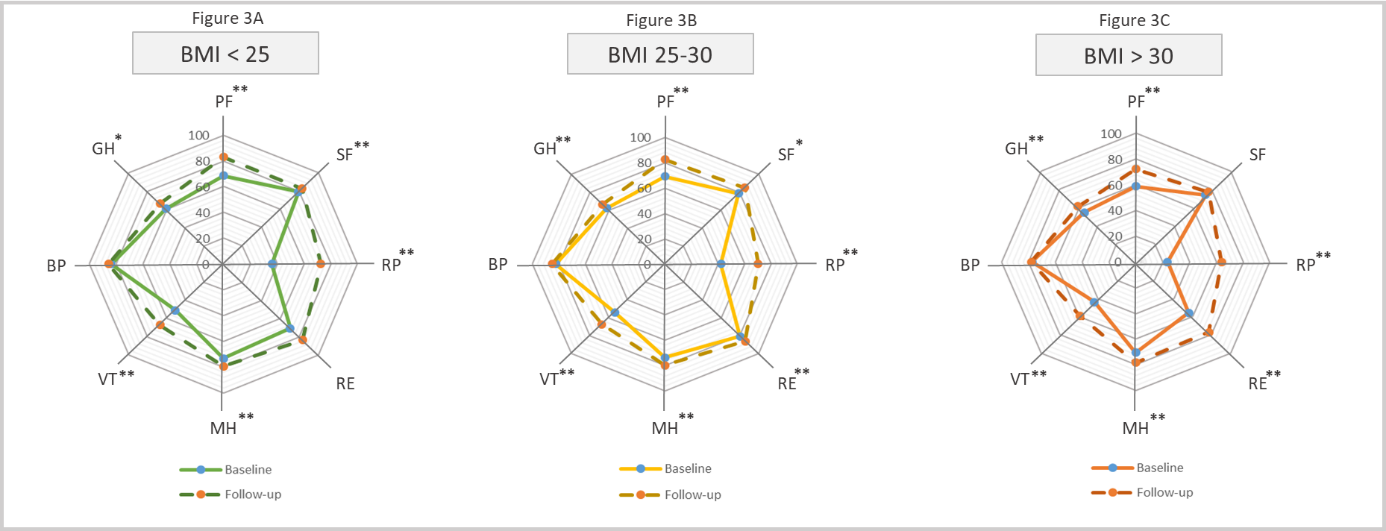


Supplementary Figure 1. Radar charts representing 8 QoL-domains at baseline and follow-up in normal weight (1a), overweight (1b) and obese (1c) patients. The center of the Radar chart displays a QoL-score of 0 (=low) and the outer edge a score of 100 (=high). * p<0.05. ** p<0.01. P-value represents significant difference in QoL-score between baseline and follow-up. For definitions of each domain please refer to “definitions” in the “Methods” section.

1c)

1b)

1a)

**Supplementary Figure 2: The relationship between BMI at baseline and the change in the physical component summary (PCS) score between the baseline and follow-up visit**

**
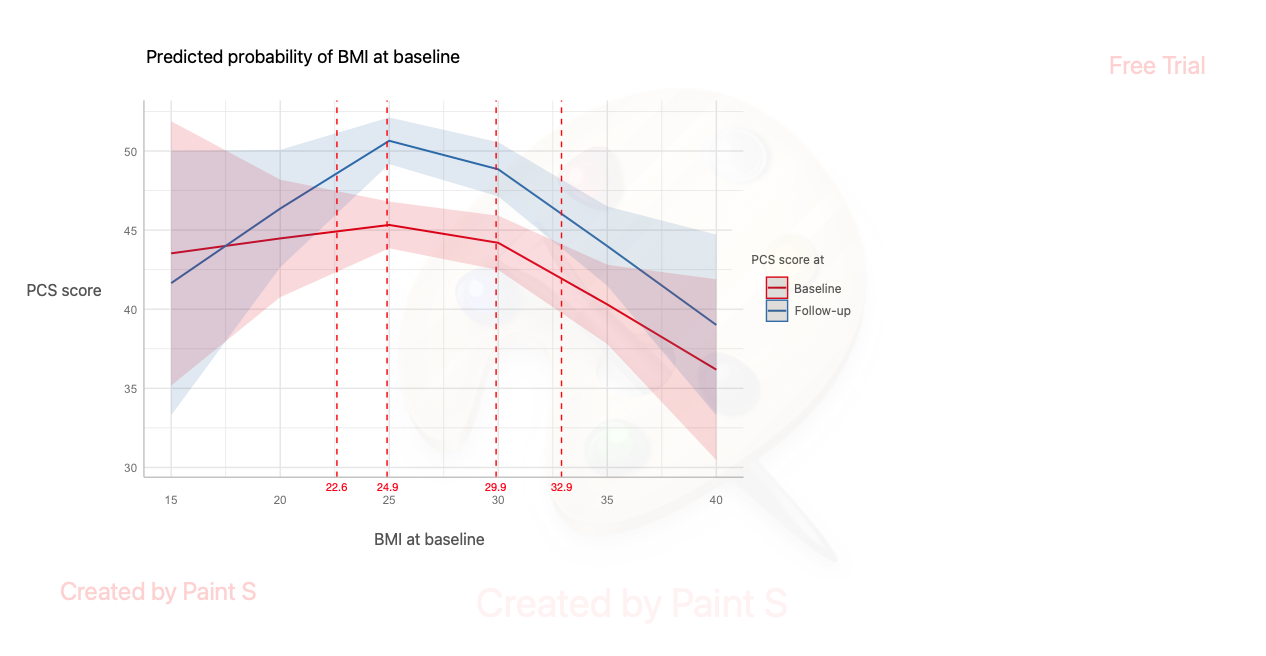
**

Supplementary Figure 2. Graph shows the relation between BMI at baseline and the course of PCS between baseline (red line) and follow-up (blue line). The 4 dotted red lines indicate the knot locations of the spline function.

##### **Supplementary Table 1: Quality of life before and after thoracoscopic AF ablation for normal, overweight and obese patients**

| **QoL at** **Baseline**  (mean±SE) | **Total group** | BMI <25 | BMI 25-30 | **p-value**  BMI <25 vs. 25-30 | BMI ≥30 | **p-value**  BMI <25 vs. ≥30 | **p-value**  BMI 25-30 vs. ≥30 | **p-value** between groups |
| --- | --- | --- | --- | --- | --- | --- | --- | --- |
| Physical functioning | 66.3±1.2 | 69.1±2.1 | 64.5±1.6 | 0.71 | 58.7±2.5 | **<0.01** | **<0.01** | **<0.01** |
| Social functioning | 77.8±1.9 | 78.8±3.5 | 79.3±2.7 | 0.94 | 73.6±3.8 | 0.32 | 0.29 | 0.51 |
| Role physical | 35.0±2.1 | 42.2±4.0 | 36.3±3.1 | 0.21 | 23.7±3.5 | **<0.01** | **0.03** | **<0.01** |
| Role emotional | 72.7±2.0 | 80.4±3.3 | 70.3±3.0 | **0.03** | 56.0±4.4 | **<0.01** | **<0.01** | **<0.01** |
| Mental Health | 49.9±0.8 | 73.6±1.5 | 73.2±1.2 | 0.84 | 70.4±1.7 | 0.15 | 0.20 | 0.31 |
| Vitality | 81.9±1.0 | 53.7±1.9 | 50.7±1.5 | 0.19 | 44.0±1.9 | **<0.01** | **<0.01** | **<0.01** |
| Bodily Pain | 59.6±1.1 | 82.5±2.1 | 83.8±1.5 | 0.73 | 77.4±2.3 | **0.03** | **<0.01** | **0.02** |
| General Health | 33.8±0.9 | 62.2±1.8 | 60.6±1.3 | 0.35 | 54.5±1.8 | **<0.01** | **<0.01** | **<0.01** |
| **Physical Component Summary** | 44.3±0.5 | 44.8±0.8 | 45.3±0.6 | 0.66 | 41.7±1.0 | **0.02** | **<0.01** | **<0.01** |
| **Mental Component Summary** | 45.7±0.6 | 47.7±1.0 | 45.9±0.8 | 0.27 | 43.1±1.2 | **<0.01** | **0.05** | **0.02** |
| **QoL at Follow-up**  (mean±SE) | **Total group** | BMI <25 | BMI 25-30 | **p-value**  BMI <25 vs. 25-30 | BMI ≥30 | **p-value**  BMI <25 vs. ≥30 | **p-value**  BMI 25-30 vs. ≥30 | **p-value** between groups |
| Physical functioning | 80.4±1.1 | 82.7±1.6 | 83.0±1.4 | 0.41 | 72.2±2.7 | **0.02** | **<0.01** | **<0.01** |
| Social functioning | 82.0±1.1 | 85.2±1.9 | 82.8±1.5 | 0.23 | 76.7±2.5 | **<0.01** | 0.05 | **0.03** |
| Role physical | 69.9±2.0 | 70.3±3.6 | 72.5±2.8 | 0.56 | 64.1±4.3 | 0.31 | 0.11 | 0.26 |
| Role emotional | 82.6±1.7 | 85.8±2.8 | 83.4±2.3 | 0.52 | 77.2±4.0 | 0.14 | 0.30 | 0.32 |
| Mental Health | 79.2±0.8 | 79.8±1.6 | 79.4±1.2 | 0.65 | 78.3±1.6 | 0.40 | 0.53 | 0.67 |
| Vitality | 65.2±1.0 | 67.3±1.8 | 67.1±1.4 | 0.86 | 59.0±2.2 | **<0.01** | **<0.01** | **<0.01** |
| Bodily Pain | 83.8±1.0 | 85.1±1.8 | 85.9±1.3 | 0.74 | 77.9±2.5 | **0.04** | **0.01** | **0.03** |
| General Health | 65.2±1.0 | 66.6±1.9 | 66.6±1.5 | 0.81 | 60.9±2.1 | 0.07 | **0.02** | 0.06 |
| Health change^x^ | 80.5±1.2 | 81.0±2.3 | 80.0±1.8 | 0.64 | 81.0±2.7 | 0.75 | 0.44 | 0.72 |
| **Physical Component Summary** | 49.3±0.5 | 50.1±0.8 | 50.3±0.6 | 0.77 | 46.3±1.1 | **0.02** | **<0.01** | **0.01** |
| **Mental Component Summary** | 50.0±0.5 | 50.8±0.9 | 49.9±0.7 | 0.29 | 49.3±1.0 | 0.34 | 0.98 | 0.51 |

Supplementary Table 1. Table describing all QoL scores; SF-domains, health change, physical component summary (PCS) and mental component summary (MCS) at baseline and follow-up; for total group and each BMI groups. P-values represent differences between groups and total group. *p<0.05; **p<0.01. All values are mean±SE. *Abbreviations:* BL = baseline. FU = follow-up at 1 year after procedure. Health change^x^: only one question regarding general change in health over 1 year, filled in at 1-year-followup.

##### **Supplementary Table 2: Quality of life change after thoracoscopic AF ablation for normal, overweight and obese patients**

| **Change in QoL** | **Total group** | | **BMI <25** | | **BMI 25-30** | | **p-value**  BMI <25 vs. 25-30 | **BMI** ≥**30** | | **p-value**  BMI <25 vs. ≥30 | **p-value**  BMI 25-30 vs. ≥30 | **p-value** between groups |
| --- | --- | --- | --- | --- | --- | --- | --- | --- | --- | --- | --- | --- |
|  | mean±SE | p-value | mean±SE | p-value | mean±SE | p-value |  | mean±SE | p-value |  |  |  |
| Physical functioning | 13.9±1.1 | **<0.01** | 13.4±2.1 | **<0.01** | 14.5±1.4 | **<0.01** | 0.49 | 13.0±2.6 | **<0.01** | 0.11 | 0.71 | 0.24 |
| Social functioning | 4.7±1.8 | **<0.01** | 7.5±3.3 | **<0.01** | 3.5±2.7 | **<0.01** | 0.18 | 3.6±3.6 | 0.09 | 0.11 | 0.71 | 0.24 |
| Role physical | 34.8±2.5 | **<0.01** | 27.8±4.8 | **<0.01** | 36.4±3.6 | **<0.01** | 0.20 | 40.2±5.1 | **<0.01** | 0.12 | 0.57 | 0.25 |
| Role emotional | 12.6±2.2 | **<0.01** | 4.9±3.8 | 0.19 | 12.6±3.1 | **<0.01** | 0.07 | 22.0±5.0 | **<0.01** | **<0.01** | 0.10 | **0.01** |
| Mental Health | 6.6±0.7 | **<0.01** | 6.3±1.3 | **<0.01** | 6.1±1.1 | **<0.01** | 0.58 | 7.6±1.8 | **<0.01** | 0.44 | 0.22 | 0.45 |
| Vitality | 15.5±1.0 | **<0.01** | 13.9±1.8 | **<0.01** | 16.6±1.4 | **<0.01** | 0.60 | 15.0±2.3 | **<0.01** | 0.51 | 0.99 | 0.81 |
| Bodily Pain | 1.5±1.1 | 0.10 | 2.6±1.9 | 0.18 | 1.3±1.5 | 0.30 | 0.49 | 0.8±2.7 | 0.61 | 0.64 | 0.98 | 0.79 |
| General Health | 5.7±0.9 | **<0.01** | 4.5±1.8 | **0.03** | 6.0±1.3 | **<0.01** | 0.37 | 6.7±2.2 | **<0.01** | 0.41 | 0.89 | 0.61 |
| **Physical Component Summary** (PCS) | 4.7±0.5 | **<0.01** | 5.0±0.8 | **<0.01** | 4.7±0.6 | **<0.01** | 0.79 | 4.4±1.0 | **<0.01** | 0.62 | 0.75 | 0.88 |
| *Relative change in PCS* | 0.6±1.3 |  | 0.6±2.3 |  | 0.4±1.7 |  | 0.64 | 0.8±2.9 |  | 0.84 | 0.79 | 0.89 |
| **Mental Component Summary** (MCS) | 4.2±0.6 | **<0.01** | 3.2±1.0 | **<0.01** | 3.9±0.8 | **<0.01** | 0.72 | 6.1±1.2 | **<0.01** | 0.09 | 0.14 | 0.20 |
| *Relative change in MCS* | 0.6±1.7 |  | 0.6±2.6 |  | 0.6±2.4 |  | 0.66 | 0.8±4.3 |  | 0.08 | 0.13 | 0.19 |

Supplementary Table 2 represents the change in QoL in total group and BMI groups. P-values per BMI group show significance of change in QoL-score. White p-values show differences in QoL-change compared between groups. Grey p-values represent change in QoL compared in the total group.

All values are mean±SE or p-values. All p-values in **bold** are significant (p<0.05).

##### **Supplementary Table 3: Quality of life changes in patients with or without AF recurrence, for obese and non-obese patients**

| **A: Total group: recurrence vs freedom of atrial fibrillation.** | | | | | | |
| --- | --- | --- | --- | --- | --- | --- |
|  | | Change in QoL | | | | |
|  |  | **AF Recurrence**  (mean±SE) | Baseline vs. Follow-up  (p-value) | **Freedom of AF**  (mean±SE) | Baseline vs. Follow-up  (p-value) | Recurrence vs Freedom of AF  (p-value) |
| **SF-domains** | PF | 7.8±2.1 | **<0.01** | 16.8±1.3 | **<0.01** | **<0.01** |
|  | SF | 0.0±3.1 | 1.0 | 6.5±2.2 | **<0.01** | **0.03** |
|  | RP | 20.7±4.6 | **<0.01** | 40.5±3.0 | **<0.01** | **<0.01** |
|  | RE | 7.7±4.0 | 0.05 | 15.2±2.6 | **<0.01** | 0.14 |
|  | MH | 3.2±1.4 | **0.02** | 7.8±0.9 | **<0.01** | **<0.01** |
|  | VT | 10.6±1.7 | **<0.01** | 17.2±1.2 | **<0.01** | **<0.01** |
|  | BP | -2.3±2.1 | 0.26 | 3.0±1.3 | **0.02** | **0.02** |
|  | GH | -2.9±1.6 | 0.08 | 9.5±1.1 | **<0.01** | **<0.01** |
| **Summary scores** | PCS | 1.6±0.8 | **0.05** | 6.0±0.5 | **<0.01** | **<0.01** |
|  | MCS | 2.8±1.0 | **<0.01** | 4.8±0.7 | **<0.01** | 0.07 |
| **Health change^x^** | | 67.6±3.1 |  | 85.9±1.8 |  | **<0.01** |

| **B: Normal weight group (BMI <25): recurrence vs freedom of atrial fibrillation.** | | | | | | |
| --- | --- | --- | --- | --- | --- | --- |
|  | | Change in QoL | | | | |
|  |  | **AF Recurrence**  (mean±SE) | Baseline vs. Follow-up  (p-value) | **Freedom of AF**  (mean±SE) | Baseline vs. Follow-up  (p-value) | Recurrence vs Freedom of AF  (p-value) |
| **SF-domains** | PF | 3.6±3.9 | 0.36 | 17.7±2.3 | **<0.01** | **<0.01** |
|  | SF | 3.6±5.5 | 0.51 | 8.7±4.0 | **0.04** | 0.25 |
|  | RP | 5.7±8.7 | 0.51 | 35.9±5.6 | **<0.01** | **<0.01** |
|  | RE | -3.3±6.7 | 0.62 | 8.5±4.6 | 0.06 | 0.15 |
|  | MH | 1.5±2.8 | 0.58 | 8.0±1.3 | **<0.01** | **0.05** |
|  | VT | 9.5±3.3 | **<0.01** | 15.3±2.1 | **<0.01** | 0.17 |
|  | BP | -1.2±3.4 | 0.73 | 4.1±2.2 | 0.08 | 0.38 |
|  | GH | -6.3±2.9 | **0.04** | 8.5±2.1 | **<0.01** | **<0.01** |
| **Summary scores** | PCS | 1.1±1.5 | 0.49 | 6.5±1.0 | **<0.01** | **<0.01** |
|  | MCS | 1.0±2.0 | 0.60 | 4.0±1.1 | **<0.01** | 0.21 |
| **Health change^x^** | | 68.8±6.8 |  | 85.4±3.3 |  |  |

| **D: Obese group (BMI ≥** **30): recurrence vs freedom of atrial fibrillation.** | | | | | | |
| --- | --- | --- | --- | --- | --- | --- |
|  | | Change in QoL | | | | |
|  |  | **AF Recurrence**  (mean±SE) | Baseline vs. Follow-up  (p-value) | **Freedom of AF**  (mean±SE) | Baseline vs. Follow-up  (p-value) | Recurrence vs Freedom of AF  (p-value) |
| **SF-domains** | PF | 8.8±5.5 | 0.12 | 15.1±3.0 | **<0.01** | 0.29 |
|  | SF | -1.4±7.6 | 0.86 | 5.0±4.1 | 0.23 | 0.77 |
|  | RP | 37.0±9.9 | **<0.01** | 41.5±6.0 | **<0.01** | 0.67 |
|  | RE | 28.2±10.7 | **0.01** | 20.7±5.6 | **<0.01** | 0.24 |
|  | MH | 3.7±3.6 | 0.31 | 8.9±2.0 | **<0.01** | 0.23 |
|  | VT | 11.5±4.3 | **0.01** | 15.6±2.7 | **<0.01** | 0.31 |
|  | BP | -2.2±6.1 | 0.72 | 2.1±3.0 | 0.50 | 0.71 |
|  | GH | -2.6±3.9 | 0.51 | 10.7±2.5 | **<0.01** | **<0.01** |
| **Summary scores** | PCS | 1.2±1.9 | 0.53 | 5.8±1.2 | **<0.01** | **0.05** |
|  | MCS | 6.3±2.4 | **0.02** | 5.8±1.4 | **<0.01** | 0.78 |
| **Health change^x^** | | 63.0±7.4 |  | 88.0±3.8 |  |  |

| **C: Overweight group (BMI 25-30): recurrence vs freedom of atrial fibrillation.** | | | | | | |
| --- | --- | --- | --- | --- | --- | --- |
|  | | Change in QoL | | | | |
|  |  | **AF Recurrence**  (mean±SE) | Baseline vs. Follow-up  (p-value) | **Freedom of AF**  (mean±SE) | Baseline vs. Follow-up  (p-value) | Recurrence vs Freedom of AF  (p-value) |
| **SF-domains** | PF | 9.4±2.5 | **<0.01** | 17.0±1.7 | **<0.01** | **<0.01** |
|  | SF | -1.2±4.1 | 0.77 | 5.8±3.4 | 0.09 | **0.04** |
|  | RP | 21.3±6.2 | **<0.01** | 42.9±4.3 | **<0.01** | **<0.01** |
|  | RE | 4.4±4.8 | 0.36 | 16.5±3.9 | **<0.01** | **0.03** |
|  | MH | 3.8±1.6 | **0.02** | 7.2±1.3 | **<0.01** | **0.03** |
|  | VT | 10.9±2.2 | **<0.01** | 19.2±1.7 | **<0.01** | **<0.01** |
|  | BP | -2.9±2.5 | 0.24 | 2.8±1.8 | 0.11 | **0.02** |
|  | GH | -1.2±2.2 | 0.59 | 9.4±1.5 | **<0.01** | **<0.01** |
| **Summary scores** | PCS | 2.0±1.0 | 0.06 | 5.9±0.8 | **<0.01** | **<0.01** |
|  | MCS | 2.1±1.3 | 0.11 | 4.8±1.0 | **<0.01** | 0.06 |
| **Health change^x^** | | 69.0±3.7 |  | 85.2±2.6 |  | **<0.01** |

Supplementary Table 3. Tables display change in QoL in total group (a) and BMI groups (b-d) divided by AF status. P-values in described after AF recurrence and freedom of AF describe significance of change in QoL in that group. All values are mean±SE or p-values. All p-values in **bold** are significant (p<0.05).

Health change^x^: only one question regarding general change in health over 1 year, filled in at 1-year-followup.

##### **Supplementary Table 4: The effect of different determinants on the change in physical and mental components of the summary scores between the baseline and follow-up visit as estimated with linear mixed models**

Linear mixed models

We used linear mixed models to estimate the effect of different determinants on the change in PCS or MCS between the baseline and follow-up visit. In each model we used a by-patient random intercept, a first order autoregressive correlation structure (by-time and by-patient), and an interaction term between the time variable (i.e., baseline or follow-up visit) and the determinant of interest. Such determinants were considered potentially meaningful if the p-value of the interaction term was <0.05. All models were fitted with the maximum likelihood method. If a model did not converge, we tried several optimizers to reassess if the model converged, and if unsuccessful, increased the number of iterations. All models converged using these methods.

For continuous determinants, we assessed if their relationship with the outcome of interest was nonlinear using a restricted cubic spline function (47, 48). The fit of more complex nonlinear relationships were assessed by iteratively increasing number of knots of the spline function. Knot locations were determined solely by the number of knots (i.e., 10^th^, 50^th^ and 90^th^ percentiles). To assess the nonlinear relationship between BMI and the course of the outcomes over time, we used the 10th and 90^th^ percentiles as the outer knot locations and the two frontiers of the BMI groups as the two inner knot locations. The model with the spline function was selected only if the ANOVA test comparing the nested models produced a p-value <0.05. Whenever the nonlinear model was the better fit, we used line plots to illustrate the nonlinear relationship between the determinant of interest and the change in the outcome of interest between the baseline and follow-up visit.

References

42. Harrell FE, et al. Regression Modeling Strategies: With Applications to Linear Models, Logistic and Ordinal Regression, and Survival Analysis. Springer International Publising. 2015; 13-44

43. Durrleman S, Simon R. Flexible Regression Models with Cubic Splines. Stat Med. 1989;8;551-61.

| **Determinants** | **PCS** | | **MCS** | |
| --- | --- | --- | --- | --- |
|  | Beta coefficient | p-value | Beta coefficient | p-value |
| BMI | *Graph** | NS | 0.29 | NS |
| 1 year freedom of AF |  |  |  |  |
| - No | Ref | Ref | Ref | Ref |
| - Yes | 4.89 | <0.01 | 1.98 | NS |
| Clinical AF pattern |  |  |  |  |
| - Paroxysmal | Ref | Ref | Ref | Ref |
| - Persistent | 0.11 | NS | -0.82 | NS |
| AF duration | -0.11 | NS | -0.10 | NS |
| History of myocard infarction | -1.08 | NS | 0.88 | NS |
| CHA2DS2-VASC score |  |  |  |  |
| - Score = 0 | 2.09 | 0.043 | -2.43 | NS |
| - Score = 1 | -0.33 | NS | -1.00 | NS |
| - Score > 1 | -1.31 | NS | 3.89 | 0.011 |
| Risk factors |  |  |  |  |
| - Age | -0.07 | NS | -0.00 | NS |
| - Female | -0.85 | NS | 0.62 | NS |
| - History of congestive heart disease | 2.75 | NS | -2.07 | NS |
| - Hypertension | -1.41 | NS | 3.02 | <0.01 |
| - DM | -3.66 | NS | 6.21 | 0.031 |
| - History of stroke | -1.39 | NS | 0.94 | NS |
| - History of vascular disease | -0.15 | NS | 1.94 | NS |
| Total number of anti-arrhytmic medication at baseline | 0.00 | NS | 2.51 | <0.01 |
| Heartrate | *Graph** | NS | 0.04 | NS |
| Pro-BNP | 0.00 | <0.01 | 0.00 | NS |

Supplementary Table 4 displays determinants of the change in physical (PCS) and mental component summary (MCS) scores between the baseline and follow-up visit.

*Abbreviations:* AF = atrial fibrillation – BMI = body mass index – CHADsVASc = score to predict thromboembolic risk (Congestive heart failure, Hypertension, Age, Diabetes, Stroke) – MI = myocardial infarction

*Graph: interaction terms are not interpretable individually, therefore use of a graph is required.

##### **Supplementary Table 5: Analysis excluded patients due to not returned QoL forms**

**Table 5a: Comparison of baseline data**

| **Baseline data** | **BMI <25** | | | | Difference in data? | **BMI 25-30** | | | | Difference in data? | **BMI ≥30** | | | | Difference in data? |
| --- | --- | --- | --- | --- | --- | --- | --- | --- | --- | --- | --- | --- | --- | --- | --- |
|  | Original data | | Excluded patients | |  | Original data | | Excluded patients | |  | Original data | | Excluded patients | |  |
|  | Mean | SE | Mean | SE | p-value | Mean | SE | Mean | SE | p-value | Mean | SE | Mean | SE | p-value |
| **PCS** | 44.8 | 0.8 | 42.9 | 2.2 | 0.47 | 45.3 | 0.6 | 43.7 | 1.2 | 0.11 | 41.7 | 1.0 | 38.2 | 2.0 | 0.15 |
| **MCS** | 47.7 | 1.0 | 43.6 | 2.4 | 0.19 | 45.9 | 0.8 | 45.8 | 1.4 | 0.88 | 43.1 | 1.2 | 39.7 | 2.2 | 0.18 |

**Table 5b: Comparison of follow-up data**

| **Follow-up data** | **BMI <25** | | | | Difference in data? | **BMI 25-30** | | | | Difference in data? | **BMI ≥30** | | | | Difference in data? |
| --- | --- | --- | --- | --- | --- | --- | --- | --- | --- | --- | --- | --- | --- | --- | --- |
|  | Original data | | Excluded patients | |  | Original data | | Excluded patients | |  | Original data | | Excluded patients | |  |
|  | Mean | SE | Mean | SE | p-value | Mean | SE | Mean | SE | p-value | Mean | SE | Mean | SE | p-value |
| **PCS** | 50.1 | 0.8 | 50.1 | 2.0 | 0.88 | 50.3 | 0.6 | 48.1 | 1.8 | 0.41 | 46.3 | 1.1 | 47.0 | 2.3 | 0.70 |
| **MCS** | 50.8 | 0.9 | 50.6 | 2.2 | 0.99 | 49.9 | 0.7 | 48.4 | 1.7 | 0.35 | 49.3 | 1.0 | 52.2 | 1.5 | 0.43 |

Supplementary Table 5 represents the comparison between the original data versus data of patients that were excluded.

Table 5a shows QoL data at baseline of included patients vs. patients that were excluded due to missing data at 1-year follow-up.

Table 5b shows QoL data at 1-year follow-up of included patients vs. patients that were excluded due to missing data at baseline.

All values are mean±SE or p-values.
